# Diagnosis and treatment of varicella-zoster virus infection with herpetic visceral neuralgia without rash: A case report

**DOI:** 10.1097/MD.0000000000033766

**Published:** 2023-05-26

**Authors:** Jin Zeng, Yuanyuan Yang

**Affiliations:** a Department of Anesthesiology, First Affiliated Hospital of Guangxi University of Science and Technology, Liuzhou, China; b First Affiliated Hospital of Guangxi University of Science and Technology, Liuzhou, China.

**Keywords:** diagnostic therapy, herpes zoster neuralgia, herpetic visceral neuralgia, postherpetic neuralgia, varicella-zoster virus

## Abstract

Herpes zoster (HZ) is an infection caused by the varicella-zoster virus reactivation, often leading to peripheral nervous system infection and pain. This case report aimed to present 2 patients with damaged sensory nerves originating from the visceral neurons of the lateral horn of the spinal cord.

**Patient concerns:**

Two patients presented intractable, severe lower back pain and abdominal pain, but without rash or herpes. A female patient was admitted 2 months after symptom onset. She was presented with paroxysmal, acupuncture-like pain in the right upper quadrant and around the umbilicus without apparent incentives. A male patient was presented with recurrent episodes of paroxysmal and spastic colic in the left waist and left middle abdomen for 3 days. Abdominal examination showed no tumors or organic lesions in their intra-abdominal tissues or organs.

**Diagnoses:**

After excluding organic lesions on the waist and in abdominal organs, patients were diagnosed with herpetic visceral neuralgia without rash.

**Intervention:**

The treatment for herpes zoster neuralgia or postherpetic neuralgia was applied for 3 to 4 weeks.

**Outcome:**

Antibacterial and anti-inflammatory analgesics were not effective in either patient. The therapeutic effects of herpes zoster neuralgia or postherpetic neuralgia treatment were satisfactory.

**Lessons:**

Herpetic visceral neuralgia can be easily misdiagnosed due to the absence of a rash or herpes, resulting in delayed treatment. When patients have severe, intractable pain but no rash or herpes, and the biochemical and imaging examinations are normal, the treatment method for HZ neuralgia can be used. If the treatment is effective, HZ neuralgia is diagnosed. If not, shingles neuralgia can be ruled out. Further investigations are required to elucidate the mechanisms of pathophysiological changes in varicella-zoster virus-induced peripheral HZ neuralgia or visceral neuralgia without herpes.

## 1. Introduction

Herpes zoster (HZ) is an infection caused by the reactivation of the varicella-zoster virus (VZV). It commonly occurs in patients with severe diseases of the heart, lung, brain, liver, or kidney, with malignant tumors, or undergoing immunosuppressive therapy. VZV reactivation in the sensory ganglia leads to peripheral nervous system infection, which subsequently causes a band of rash and spontaneous pricking and burning sensations.^[1]^ More than 90% of the cases with HZ have a concomitant occurrence of erythema, papules, small vesicles, or blood herpes on the skin innervated with sensory nerve endings and are accompanied by spontaneous sensations of burning, pricking, cutting, electric shock, throbbing, numbness, and itching, as well as hyperalgesia.^[2–4]^

Very few patients have shingles neuralgia accompanied by skin sensation or hyperalgesia but no rash. It is called rash-free shingles and can be treated as cases with shingles. However, when VZV invades the splanchnic neurons/splanchnic nerve fibers and causes pain in visceral organs, it is characterized by a wide range of pain and inaccurate localization on the body surface. As the skin sensory nerves are not involved, there is only intractable, paroxysmal colic, acupuncture, or distending pain in the chest or abdominal cavities, especially at night.

In this case report, we aimed to present 2 patients with damaged sensory nerves originating from the visceral neurons of the lateral horn of the spinal cord. They showed no clinical symptoms or imaging features of visceral neuralgia, making the VZV diagnosis challenging. The treatment for HZ neuralgia was applied to the patients.

## 2. Case presentation

Case 1: A 55-year-old female patient experienced spontaneous pricking and burning sensations and severe pain in the right upper quadrant and around the umbilicus for 2 months without obvious incentives. The Visual Analogue Scale (VAS) score was 7 to 8 at admission and was 4 to 5 in remission. The pain was more severe at night. The patient had no nausea, vomiting, acid reflux, belching, chills, fever, cough, expectoration, diarrhea, or constipation, and the urine was normal. After the onset of symptoms, the patient was treated for hepatobiliary disease and acute gastroenteritis in other hospitals. The medications included oral amoxicillin capsules for antibacterial infection, diclofenac sodium enteric-coated tablets for anti-inflammation and analgesia, and rabeprazole to inhibit gastric acid secretion; however, none of them was effective. The patient had a Pittsburgh Sleep Quality Index (PSQI) score of 17, indicating poor sleeping quality. Oral tramadol sustained-release tablets (100 mg) were ineffective when the patient had severe pain. The pain was significantly relieved 15 minutes after intramuscular injection of 100 mg of tramadol or 50 mg of pethidine, with a VAS score of 0 to 1. After each intramuscular injection of analgesics, analgesia was maintained for approximately 3 hours. Then, severe pain recurred, with a VAS score of 7 to 8. The pain could only be relieved by repetitive intramuscular injections of tramadol or pethidine hydrochloride. The patient suffered from nausea, vomiting, dizziness, and poor appetite after using analgesics. She was also distressed and depressed.

After being admitted to our hospital, the patient did not develop a rash since the onset of symptoms. Physical examination showed no signs of skin erythema, rash, herpes, or skin pigmentation on the head, neck, chest, back, waist, abdomen, or limbs. There was no tenderness in the intervertebral, thoracic paravertebral, lumbar paravertebral, or thoracic intercostal spaces. The patient experienced tenderness in the right upper quadrant, the subxiphoid process, and the umbilicus. There was no paresthesia or hyperalgesia at the site of the pain. Fiberoptic gastroscopy showed that the structures of the esophagus, fundus, antrum, gastric angle, pylorus, duodenal bulb, and the descending part were normal. In the lesser curvature of the upper gastric body, a 0.3 × 0.2 cm mucosal concave and whitish structure was observed. Three polyps (0.2 × 0.1 × 0.1 cm) were also detected. There was no erosion or tumor growth in other sites. Histopathological biopsy showed moderately active gastritis in the gastric body, mucosal erosion, and inflammatory polyps. The 64-slice computed tomography (CT) with 3-dimensional imaging of the whole abdomen (including the stomach) showed no abnormality in the liver, gallbladder, stomach, pancreas, spleen, appendix, kidneys, calyces, ureter, bladder, abdominal aorta, abdominal cavity, or retroperitoneal cavity. The coagulation function was normal. The D-dimer was 0.58 µg/mL. Electrocardiogram and coronary CT angiography showed no abnormality. The blood, stool, and urine were also normal.

This patient underwent gastroscopy, during which several gastric polyps were removed. However, the pain in the right upper quadrant and the subxiphoid gastric was not relieved after the operation. Multidisciplinary treatment was performed after 4 days of hospitalization. Although this patient had recurrent, spontaneous, burning, acupuncture-like paroxysmal pain for 8 weeks, laboratory examinations suggested no organic diseases in the abdomen, chest, spine, or spinal canal and no pain caused by cardiopulmonary diseases. The above examination results suggest that the visceral pain might be caused by HZ infection of the splanchnic nerve. Therefore, the treatment for shingles neuralgia was applied. The following medications were given to the patient: oral gabapentin, 300 mg/time, 3 times per day (t.i.d); pregabalin capsules, 75 mg, every night (q.n.); methylcobalamin tablets, 1.0 mg, t.i.d; vitamin B1, 20 mg, times per day (t.i.d.) Tramadol was discontinued on the treatment day. The pain was significantly relieved after 12 hours of treatment, with a VAS score of 2 and a PSQI score of 7, which significantly improved compared to those before treatment. On the second day of treatment, the VAS score was 1, and the PSQI score was 6. On the third day of treatment, the VAS score was 0 to 1, and the PSQI score was 5. After 7 days of treatment, the patient stopped taking gabapentin and pregabalin capsules. That night, she developed subxiphoid burning pain in the upper abdomen, with a VAS score of 5. The patient was immediately given 300 mg of gabapentin and 75 mg of pregabalin. The pain was significantly relieved 2 hours later, with a VAS score of 1 and a PSQI score of 5. After 2 weeks of treatment, the VAS score of the patient was 0, and the PSQI score was 3. All medications were discontinued after 3 weeks of treatment. The patient had no abdominal pain, and the sleep quality was improved. She experienced no pain during 2 months of follow-up.

Case 2: A 66-year-old male patient was admitted to our hospital with recurrent episodes of paroxysmal and spastic colic in the left waist and left middle abdomen for 3 days for unknown reasons. He had a history of type 2 diabetes and took oral hypoglycemic medications to maintain fasting blood glucose levels of 6.0 to 8.0 mmol/L and glycated hemoglobin levels of 10.5%. The pain lasted 2 to 4 hours after the onset and recurred 2 hours after the relief. The VAS score was 7 to 8 when the pain occurred and was 4 when the pain was relieved. The pain severely affected the patient’s sleep quality, with a PSQI score of 17. Ureteral calculi were considered the cause of the pain at admission. The patient was given analgesics (i.e., tramadol or pethidine) either orally or via intramuscular injection to relieve spasms. The pain was relieved after an intramuscular injection of pethidine.

Physical examination showed no signs of skin rash or herpes. There was no percussion pain in the bilateral renal regions. The entire abdomen was soft, and the mass was not palpable. The deep left lower quadrant tenderness was evident, with no rebound tenderness. There was no tenderness at McBurney point or in the right abdomen. The color Doppler ultrasound of the abdomen showed a normal liver, gallbladder, pancreas, spleen, kidneys, adrenal glands, ureters, and bladder. The blood, urine, and stool, as well as liver and kidney function, were normal. The 64-slice CT of the whole abdomen showed that the gastrointestinal tract, mesentery, appendix, retroperitoneum, abdominal aorta, inferior vena cava, intraperitoneal liver and biliary tract, pancreas, and spleen were normal. Also, there was no sign of a tumor in the abdominal cavity or intra-abdominal organs.

The patient showed no rash or herpes on the body on the 6th day of admission. According to the characteristics of the pain and examination results, this patient might have herpetic visceral neuralgia. He was given the following medications: oral acyclovir tablets, 0.4 g, 4 t.i.d. for 7 days; oral gabapentin tablets, 300 mg/time, t.i.d; oral pregabalin capsules, 75 mg/time, q.n.; methylcobalamin tablets, 1.0 mg/time, t.i.d; vitamin B1, 20 mg, t.i.d. The pain was significantly relieved at night, with a VAS score of 3 and a PSQI score of 7. After 8 days of treatment, the pain was significantly relieved, with a VAS score of 1 to 2. The sleep quality was also greatly improved. After discharge, the patient continued to take gabapentin tablets (300 mg/time, t.i.d.), pregabalin capsules (75 mg/time, q.n.), and mecobalamin tablets (1.0 mg/time, t.i.d.). After 4 weeks of treatment, the patient stopped all medication. He was completely recovered with no obvious discomfort and high-quality sleep.

## 3. Discussion

The annual incidence of HZ is 3 to 5% worldwide, and approximately 9% to 34% of the HZ patients will develop postherpetic neuralgia (PHN). The incidence and prevalence of both HZ and PHN tend to increase with age.^[2,3]^

VZV is latent in the spinal dorsal root ganglia, brain ganglia, and splanchnic neurons. In most patients, reactivation of VZV results in a banded distribution of skin erythema, blisters, or pustules on the skin innervated by sensory peripheral sensory nerves. Shingles often occur on the chest, back, head, neck, face, waist, abdomen, and upper and lower limbs. They are mostly accompanied by spontaneous needle-like, knife-like, tear-like, discharge-like, hitting-like, throbbing pain, or skin hyperalgesia and numbness.^[4]^ Therefore, HZ and PHN can be clinically diagnosed based on the pain characteristics and the appearance of rash or herpes without the results of laboratory examinations.^[5,6]^ VZV is latent in the splanchnic neurons in a small group of patients after primary infection. Reactivation of VZV in patients with low resistance or damage in the splanchnic sensory nerves leads to splanchnic neuralgia, which is different from rash or herpes in peripheral sensory nerve fibers originating from the dorsal root ganglia or cranial ganglia. Visceral sensory neurons originate from the lateral horn of the spinal cord. The characteristics of visceral neuralgia and splanchnic neuralgia are different. Injuries to splanchnic nerve function may cause dull pain, referred pain, slowed gastrointestinal motility, and endocrine abnormalities, while somatic nerve injury may cause motor dysfunction and body surface pain. When VZV invades the visceral neurons and damages the sensory nerve fibers that innervate the visceral organs, intractable visceral pain may be caused. It is difficult for patients to localize the pain, which may lead to misdiagnosis and mistreatment of herpetic visceral neuralgia and postherpetic visceral neuralgia, eventually imposing a heavy economic burden on patients and resulting in a waste of medical resources.

Many treatments are available for patients with shingles or postherpetic neuralgia. However, the effects vary from person to person. Early treatment methods include antiviral agents, analgesics for pathological neuropathy, nutritional medicine for nerve rehabilitation, nerve block, minimally invasive nerve intervention (i.e., spinal cord stimulation, dorsal sensory ganglion radiofrequency pulse therapy, etc), O_3_, and traditional Chinese medicine. VZV vaccination has been considered an essential preventive treatment for HZ and PHZ in the population over 50 years old.^[7]^ The 2 patients presented here had severe and intractable pain before diagnostic treatment. We first excluded organic lesions on the site of the pain and in adjacent tissues or organs. Then, considering the characteristics and degree of the pain, the patient’s age, and combined primary diseases, the diagnosis of herpetic visceral neuralgia or postherpetic visceral neuralgia was made.^[8]^ The diagnostic treatment for HZ or PHN was applied. Case 1 had been hospitalized in our hospital for over 2 months, during which no antidote was given according to the treatment plan for PHZ. Only medications for pathological neuralgia and neurotrophic drugs were used. Case 2 had a short onset time at admission. He was given antiviral drugs for 1 week and analgesics and neurotrophic drugs during the diagnostic treatment. According to our experience, treatments for HZ or PHN with typical skin rash are complex. In some patients, the treatment effect is poor in the acute phase, and the course of the disease is prolonged. The main reason is that the pathological mechanisms of neuralgia in HZ and PHN are distinct. The therapeutic effects of the treatments on our patients were satisfactory. Further investigations into the mechanisms of the treatment are warranted.

If a patient has intractable, severe pain in the chest, waist, and abdomen without rash or herpes, and the results of physical examination, routine biochemical examination, ultrasound, and CT or magnetic resonance imaging scanning are normal, the IgG and immunoglobulin M (IgM) antibodies against VZV can be detected in serum. An increase in the IgG titer and no changes in the IgM titer indicate a history of VZV infection. An increase in both IgG and IgM titers indicates recent VZV infection, which is a diagnostic criterion for herpetic visceral neuralgia or postherpetic visceral neuralgia. As the IgM titer may increase 1 to 2 weeks after VZV infection, recent VZV infection cannot be ruled out even if the IgM titer is low.^[9]^

The limitation of the present study is that these patients were not tested for VZV antibodies before diagnostic treatment.

In conclusion, when a patient has intractable severe visceral pain but no rash or herpes, and his/her biochemical and imaging results are normal, this patient may have visceral neuralgia due to VZV infection. The levels of IgG and IgM antibodies in serum need to be measured. When the measurements for IgG and IgM titers are not available, treatments for HZ neuralgia can be applied.

## Acknowledgments

We thank our colleagues in the Department of Anesthesiology, Department of Emergency, and Department of Gastroenterology for their hard work and assistance.

## Author contributions

Conceptualization: Jin Zeng, Yuanyuan Yang.

Data curation: Jin Zeng, Yuanyuan Yang.

Formal analysis: Jin Zeng, Yuanyuan Yang.

Investigation: Jin Zeng, Yuanyuan Yang.

Methodology: Yuanyuan Yang.

Project administration: Yuanyuan Yang.

Resources: Jin Zeng, Yuanyuan Yang.

Supervision: Yuanyuan Yang.

Validation: Yuanyuan Yang.

Visualization: Yuanyuan Yang.

Writing – original draft: Jin Zeng.

Writing – review & editing: Yuanyuan Yang.

## References

[1] Jin J. Shingles vaccination. JAMA. 2018;320:416. 30043067 10.1001/jama.2018.7263

[2] Kawai K Gebremeskel BG Acosta CJ. Systematic review of incidence and complications of herpes zoster: towards a global perspective. BMJ Open. 2014;4:e004833. 10.1136/bmjopen-2014-004833 PMC4067812 24916088

[3] Zhang Y Gu XH Zhu T. Progress in the pathogenesis of spinal cord in patients with postherpetic neuralgia. Med Recapitulate. 2017;23:4256–61.

[4] Li LM Zhang ZL. Clinical observation on the treatment of postherpetic neuralgia by pulsed radiofrequency in dorsal root ganglion combined with ozone point injection. Guid J Tradit Chin Med Pharm. 2020;26:104–7.

[5] Gao CR Fan BF Lu ZH. Diagnosis and Treatment of Neuropathic Pain. Beijing: People’s Medical Publishing House; 2013:662–71.

[6] Nalamachu S Morley-Forster P. Diagnosing and managing postherpetic neuralgia. Drugs Aging. 2012;29:863–9. 23038608 10.1007/s40266-012-0014-3 PMC3693437

[7] Andrei G Snoeck R. Advances and perspectives in the management of varicella-zoster virus infections. Molecules. 2021;26:1132. 33672709 10.3390/molecules26041132 PMC7924330

[8] Henze L Buhl C Sandherr M. Management of herpesvirus reactivations in patients with solid tumours and hematologic malignancies: update of the guidelines of the infectious diseases working party (AGIHO) of the German society for hematology and medical oncology (DGHO) on herpes simplex virus type 1, herpes simplex virus type 2, and varicella zoster virus. Ann Hematol. 2022;101:491–511. 34994811 10.1007/s00277-021-04746-y PMC8810475

[9] Li R Ou M Yang S. Change in Cav3.2 T-type calcium channel induced by varicella-zoster virus participates in the maintenance of herpetic neuralgia. Front Neurol. 2021;12:741054. 34917013 10.3389/fneur.2021.741054 PMC8671009

